# Spontaneous Coronary Dissection Review: A Complex Picture

**DOI:** 10.31083/j.rcm2512448

**Published:** 2024-12-23

**Authors:** Mario Bollati, Vincenzo Ercolano, Pietro Mazzarotto

**Affiliations:** ^1^Cardiologia, Ospedale Maggiore, 26900 Lodi, Italy

**Keywords:** dissection, SCAD, myocardial infarction

## Abstract

Spontaneous coronary artery dissection (SCAD) represents a quite rare event but with potentially serious prognostic implications. Meanwhile, SCAD typically presents as an acute coronary syndrome (ACS). Despite the majority of SCAD presentation being characterized by typical ACS signs and symptoms, young age at presentation with an atypical atherosclerotic risk factor profile is responsible for late medical contact and misdiagnosis. The diagnostic algorithm is similar to that for ACS. Low-risk factors prevalence and young age would push toward non-invasive imaging (such as coronary computed tomography (CT)); instead, the gold standard diagnostic exam for SCAD is an invasive coronary angiography (ICA) due to its increased sensitivity and disease characterization. Moreover, intravascular imaging (IVI) improves ICA diagnostic performance, confirming the diagnosis and clarifying the disease mechanism. A SCAD–ICA classification recognizes four angiographic appearances according to lesion extension and features (radiolucent lumen, long and diffuse narrowing, focal stenosis, and vessel occlusion). Concerning its management, the preferred approach is conservative due to the high rates of spontaneous healing in the first months and the low rate of revascularization success (high complexity percutaneous coronary intervention (PCI) with dissection/hematoma extension risk). Revascularization is recommended in the presence of high-risk features (such as left main or multivessel involvement, hemodynamic instability, recurrent chest pain, or ST elevation). The first choice is PCI; coronary artery bypass graft (CABG) is considered only if PCI is not feasible or too hazardous according to the operators’ and centers’ experience. Medical therapy includes beta blockers in cases of ventricular dysfunction; however, no clear data are available about antiplatelet treatment because of the supposed risk of intramural hematoma enlargement. Furthermore, screening for extracardiac arthropathies or connective tissue diseases is recommended due to the hypothesized association with SCAD. Eventually, SCAD follow-up is important, considering the risk of SCAD recurrence. Considering the young age of patients with SCAD, subsequent care is essential (including psychological support, also for relatives) with the aim of safe and complete reintegration into a non-limited everyday life.

## 1. Background and General Considerations

Only a few years ago, not much was known about spontaneous coronary artery dissection (SCAD), and not much research was being conducted. Indeed, the number of articles published each year on PubMed until 2012 was roughly 15. Conversely, in 2023, there were over 151 [1]. In addition, consensus documents have been published to guide the management of SCAD [2, 3]. This increased attention on SCAD is not attributable to a rise in its incidence but to increased awareness about the disease. Indeed, in past years, a significant proportion of cases passed undiagnosed due to inadequate tools and lack of clinical suspicion. Subsequently, the implementation of advanced algorithms and devices in classic coronary disease has also helped refine diagnostic frameworks and therapeutic contexts [4].

The young age of the population most often affected by SCAD, which clinically presents as an acute coronary syndrome (ACS), as well as the conditions in which it can occur (pregnancy, postpartum) [5] have further stimulated the study of the condition, considering its potential significant implications in terms of prognosis and quality of life [6, 7, 8]. This review will address various aspects of SCAD, from epidemiology to pathophysiology, and assess the therapeutic strategies and follow-up of affected patients.

## 2. Epidemiology

As mentioned, SCAD is a more common event than it once was thought [7]. Even if the data come exclusively from observational registries and case series, with a discreet margin of uncertainty, it is estimated that approximately 1–4% of all ACS cases originate from SCAD. In some registries, SCAD seems responsible for up to 20% of ACS cases in women aged under 50 years [4, 7, 9, 10, 11, 12]. The discrepancy in estimates of SCAD incidence stems from various factors, such as the experience of the admitting center with regard to pathology. Not all ages or genders are equally affected by SCAD. Indeed, SCAD typically occurs in young women, often between the ages of 40 and 50, in the absence of risk factors for ‘classic’ (atherosclerotic) coronary artery disease [12, 13]. Overall, SCAD is responsible for at least 20% of ACS during pregnancy or postpartum [5, 14, 15, 16]. Meanwhile, considering the overlap with age-related atherosclerotic disease, SCAD in older individuals is rare.

## 3. Etiopathogenesis

SCAD is defined as a separation of the intima from the media within the layered structure of the arterial wall and leads to the formation of an intramural hematoma (IMH) and consequent compression of the true lumen, causing a reduction or even abolition of blood flow. By definition, in SCAD, the event occurs “spontaneously” in the absence of atherosclerosis and not because of medical procedures, drug effects, substance abuse, or trauma [17]. Beyond the definition of SCAD, the mechanism through which the dissection occurs remains unclear.

### 3.1 Proposed Mechanisms

Speculations about the mechanisms through which the formation and propagation of dissection occur are summarized in the following two hypotheses:

- Outside-in: a lesion in the intima (the “flap”) allows blood to infiltrate into the subintimal space, resulting in the formation of an intramural hematoma [18, 19].

- Inside-out: the formation of an intramural hematoma is the *primum movens* (likely due to rupture of the vasa vasorum), as also highlighted by intravascular ultrasound (IVUS) and optical coherence tomography (OCT) studies; the intimal lesion then becomes a consequence of the hematoma [20, 21].

Although initially underestimated, recent evidence from advancements in diagnostic imaging seems to support the second hypothesis. OCT intravascular images show how the intimal dissection or intramural hematoma is the origin of the phenomenon, even in the absence of intimal lesions [2]. Beyond the initial mechanism, the final path and clinical consequences are common to both hypotheses. The true lumen is reduced or obstructed with consequent flow impairment, either by the hematoma or the dissection flap, resulting in myocardial ischemia and necrosis distal to the affected segment [22, 23, 24]. Various triggers have been implicated (psychological, physical, hormonal, inflammatory) [7, 25]. Dissections involving multiple coronary arteries or even arteries in other districts suggest a systemic predisposition to dissection development in response to some stimuli [26]. Thus, SCAD appears to be associated, in some cases, with systemic inflammatory diseases, such as celiac disease or lupus [7, 10, 26].

### 3.2 Pathological Anatomy

Microscopic examination of an arterial wall affected by SCAD demonstrates the predominance of eosinophilic inflammatory infiltrate limited to the adventitia and peri adventitia; however, it is unclear if this finding represents the cause or the effect of the dissection [27, 28]. Differently, in arteritis, the inflammatory infiltrate also involves the media and adventitia, as in the case of polyarteritis nodosa or polyangiitis or in the case of post-COVID-19 vaccination medium vessel vasculitis [29]. Alternatively, cystic medial necrosis is not a constant finding [30]. No data regarding the evolution of chronicized SCAD in atherosclerosis are available.

## 4. Predisposing Factors and Triggers

Although knowledge about SCAD remains quite limited and mostly derived from observational registries, certain elements represent a risk factor (or predisposing factor) for the development of SCAD in the presence of an adequate trigger.

### 4.1 Hormones

Starting from the epidemiological finding of the asymmetrical distribution of SCAD, with the predominant involvement of women, it is clear how the balance of female hormones plays an important role: There are no differences between nulliparous and parous women since peripartum SCAD accounts for only 15% of cases [18, 31, 32]. Observational registries, however, reveal that peripartum SCAD has a typical distribution: often multivessel, involving the proximal segment, determining ST elevation, and compromising left ventricular systolic function, with an increased prevalence of cardiogenic shock [33].

### 4.2 Connective Diseases

Both pathological specimens and epidemiological findings have suggested a relationship between connective tissue diseases and SCAD. Nonetheless, real-world registries provide conflicting results: Analyzing the most known heritable connective tissue disorders (Loeys–Dietz syndrome, Ehlers–Danlos syndrome, Marfan’s syndrome) noted a positive correlation in only 5% of cases [34, 35]. A likely explanation may be that genetic alterations leading to connective tissue disorders are more numerous than those known. Alternatively, connective tissue disorder is not a determinant but a risk factor, although not one of the strongest. Other limited observations have hypothesized the involvement of the *TGFβ1* gene [36]. However, the supporting evidence remains limited.

### 4.3 Genetic Diagnostic

Other genes may correlate with SCAD. For example, genes such as axonemal microtubule associated, retinitis pigmentosa (*RP1*), long Intergenic non-protein coding RNA 3010 (*LINC00310*), *FBN1*, and thrombospondin repeat containing (*ADAMTSL4*) (associated with other arteriopathies), Talin1 (*TLN1*) (encoding for actin linking to the cytoskeleton of the extracellular matrix), as well as phosphatase and actin regulator 1–endotelin (*PHACTR1*–*EDN1*) (correlates with migraine, fibromuscular dysplasia, and vertebral artery dissection) [37, 38]. This genetic hypothesis could also be supported by the presence of various manifestations of arteriopathy in multiple districts, such as aneurysms or tortuosity, the so-called extra coronary vascular abnormalities (EVA) [18, 26]. For example, cerebral EVA can be diagnosed in more than 20% of patients with SCAD [39]. Therefore, it is important to defer the patient to a tertiary center for a complete evaluation [3].

### 4.4 Anatomy and Hemodynamic

Some computational experimental studies, with 3D flow reconstructions of the coronary artery (three-dimensional quantitative coronary angiography) in patients with SCAD, have shown that SCAD is more likely to occur in tortuous segments subjected to higher wall shear stress due to spatial conformation [40].

### 4.5 Atherosclerotic Risk Factors

Hypertension, dyslipidemia, and smoking are also associated with SCAD, although the mechanisms remain unclear. The only clear relation is between smoke and oxidative stress [19, 22, 41].

### 4.6 Pregnancy

Pregnancy, childbirth, and the postpartum period are significant triggering elements for SCAD: 40% of ACS during pregnancy are attributable to SCAD, with the highest SCAD risk during pregnancy or ≤30 days following delivery. Multi-parity (>4 births) seems to be a relevant risk factor for SCAD [7, 42, 43]. The reason for high SCAD susceptibility in peripartum/pregnancy is probably due to high progesterone levels, related to mucopolysaccharides and elastic fiber replacement in arterial media, with a reduction in collagen [43, 44]. In an observational registry, the prognosis of SCAD during pregnancy was found to be equal to SCAD in the general population [45].

### 4.7 Triggers/Precipitating Stressors

Several recognized stimuli play a role in SCAD, especially in the presence of predisposing factors: elevated blood pressure, physical stress (especially in males) [35], Valsalva maneuver (for example, during asthmatic crisis with repeated cough), or psychological stress (especially in females) [46]. Physical trauma can also trigger SCAD, with mechanisms likely common to physical triggers [15]. Furthermore, substance abuse may trigger SCAD [47].

## 5. Clinical Presentation

The typical presentation of SCAD is ACS, with symptoms of chest pain, nausea and vomiting, diaphoresis, syncope, headache, and shortness of breath [2, 48]. In a case series of nearly 200 patients with SCAD, it was found that chest pain was present in over 90% of cases, nausea and vomiting in 23%, dyspnea in 19%, ventricular arrhythmias in 8%, and syncope in 0.5% [49]. These data are similar in other registries [50]. Notably, over 40% of patients can be asymptomatic without persistent elektrocardiogram (ECG) abnormalities. These modalities of presentation, coupled with a low-risk profile for atherosclerotic disease, are insidious, as they can lead to an incorrect underestimation of the condition, resulting in a misdiagnosis [2, 49].

Sometimes, patients underestimate symptoms (considering themselves at low risk for ACS), leading to a delay in accessing the emergency department and subsequent delay in treatment [49]. In this context, it is paramount to consider SCAD in the differential diagnosis of ACS to minimize the effects of a potentially life-threatening condition [49, 51]. To further complicate the matter, troponin is negative in a third of cases, which is an additional significant confounding factor increasing misdiagnosis risk [51]. The differential diagnosis should also include other conditions typical in young individuals: aortic dissection, myocarditis, and pericarditis [52].

## 6. Diagnostic Modalities

The diagnostic flowchart resembles “classic” ACS, to which we refer [53]. Once a diagnosis of ACS is made, it is crucial to determine the origin of the ACS (whether it is atherosclerotic or other type). Of note, occasional detection of SCAD in stable and asymptomatic patients is extremely rare [54].

### 6.1 Non-invasive Imaging Modalities (Coronary Computed Tomography Angiography, CCTA, Cardiac Magnetic Resonance, CMR)

CCTA is reserved for low-intermediate risk patients, also in suspected SCAD. Currently, specific diagnostic criteria in SCAD are not clearly defined [54, 55, 56]. Derived from limited experience, the main CCTA findings in SCAD are (a) abrupt stenosis, (b) intramural hematoma, (c) tapered stenosis >50%, and (d) dissection flap [57]. To increase diagnostic accuracy, it is preferable to use retrospective protocol with a higher radiation dose [54]. In the case of small tortuous vessels or small distal branches, sensitivity may be low due to temporal and spatial resolution limitations. CCTA sensitivity and specificity are high (94% and 83%) in atherosclerotic disease compared to a coronary angiogram, but no data about diagnostic accuracy in SCAD are available [2]. A recent trial compared CCTA diagnostic accuracy vs. invasive angiography plus OCT in SCAD: CCTA sensitivity was less than 80%, with false negatives, especially in distal segments of SCAD [58]. Moreover, CCTA cannot discriminate between atherosclerotic disease and SCAD in vessel occlusion but can detect increased risk elements related to SCAD (such as coronary aneurysm, tortuosity, and myocardial bridge) [59]. Furthermore, CCTA can detect SCAD or atherosclerotic disease in extracardiac segments. As always, CCTA may play a relevant role in “triple rule out” (excluding aortic dissection, coronary artery disease, and pulmonary embolism) in urgent/emergent ED settings [53]. Cardiac magnetic resonance (CMR) plays a marginal role in SCAD diagnosis and definition in the acute setting but can detect motion abnormality and ischemic scarring [55, 60]. Magnetic resonance imaging (MRI) may also provide prognostic factors (microvascular obstruction, perfusion defects, left ventricular ejection fraction) [61]. The role of CCTA is prominent in the follow-up of SCAD since, in this case, the preferable diagnostic exam is certainly the non-invasive one, given the risk of propagation of residual dissection [58].

**Table:** 

**SCAD diagnostic golden rule**
All other symptoms being equal, the less likely it is a ‘classic’ ACS, the more likely it is SCAD.

### 6.2 Invasive Coronary Angiography (ICA)

Coronary angiography is the gold standard in ACS and SCAD diagnosis and treatment, even if the risk of propagating the dissection flap through catheter manipulation and contrast medium injection is considerable [22, 42]. The different findings during an ICA for SCAD may be [22]:

(1) Non-atherosclerotic appearance of the coronary arteries

(2) Dissection or separation of the coronary artery wall layers by flap

(3) Focal narrowing or irregularities

(4) Long, smooth, or tapered stenosis

(5) Tortuosity, twisting, bridging of the affected artery

(6) Contrast staining or extravasation outside the vessel lumen

(7) Normal or near-normal appearance of non-dissected coronary arteries.

ICA clarifies the clinical scenario, allowing for the identification of the ACS mechanism and high-risk anatomical features (such as thrombolysis in myocardial infarction (TIMI) flow, left main, proximal, and multivessel involvement) [35]. Furthermore, compared to other methods, ICA has the added value of delving into intracoronary imaging techniques such as IVUS and OCT by identifying intimal lesions, false lumens, and fenestrations [2, 20, 31].

The most accepted ICA-based classification is the following (Table 1 [6, 23]):

**Table 1.** **Spontaneous coronary artery dissection (SCAD) angiographic classification, modified from**.

Group	Specific features
Type 1	Dual lumen image with radiolucent dissection flap and contrast dye staining. (Fig. 1)
Type 2	A segment affected by stenosis (compression from hematoma). The stenosis may end in a mid-segment (Type 2A) or continue to the distality of the vessel (2B). Similarly called “stick insect” or “radish”. (Figs. 1,2,3)
Type 3	It mimics the appearance of complicated atherosclerotic plaque. Usually shorter than 20 mm (unlike Type 2). The mechanism is always compression from intramural hematoma. (Figs. 4,5)
Type 4	Vessel occlusion. Difficult to differentiate from embolic occlusion or atherosclerotic-based occlusion. (Fig. 6)

**Fig. 1.**
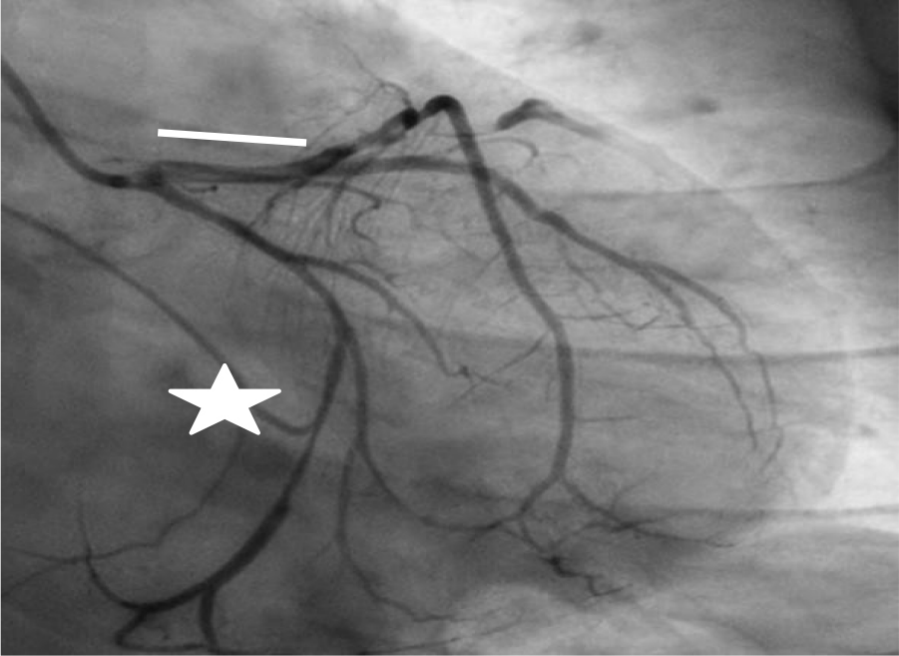
**Left main/left anterior descending Type 1 spontaneous coronary artery dissection (SCAD) (white line), circumflex Type 2A SCAD “stick insect” (white star)**. The patient went to CABG for hemodynamic instability and recurrent ST elevation. CABG, coronary artery bypass graft.

**Fig. 2.**
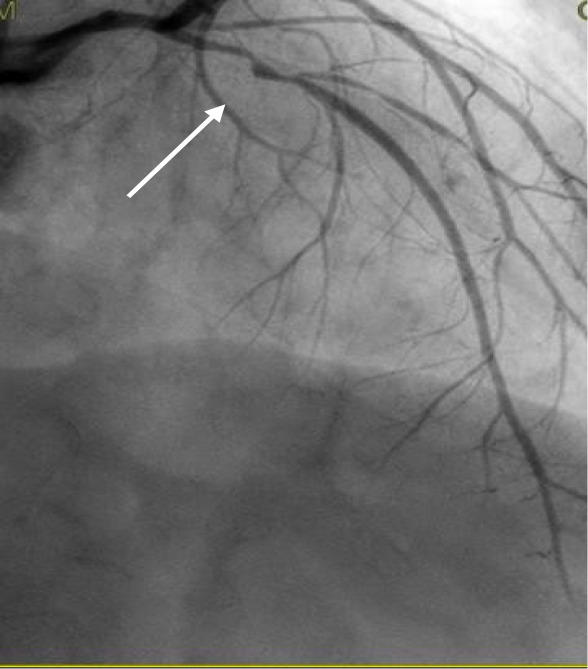
**Proximal left anterior descending Type 2A SCAD with propagation to diagonal branch (white arrow)**. SCAD, spontaneous coronary artery dissection.

**Fig. 3.**
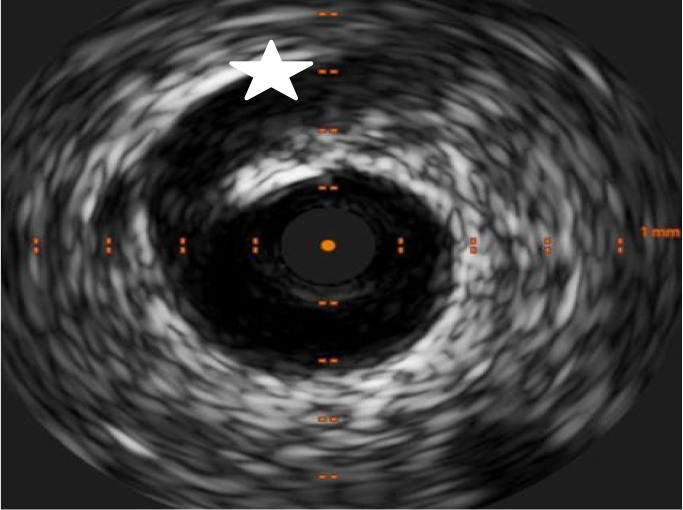
**Intimal tear (white star) and hematoma in the same patient from Fig. 2**.

**Fig. 4.**
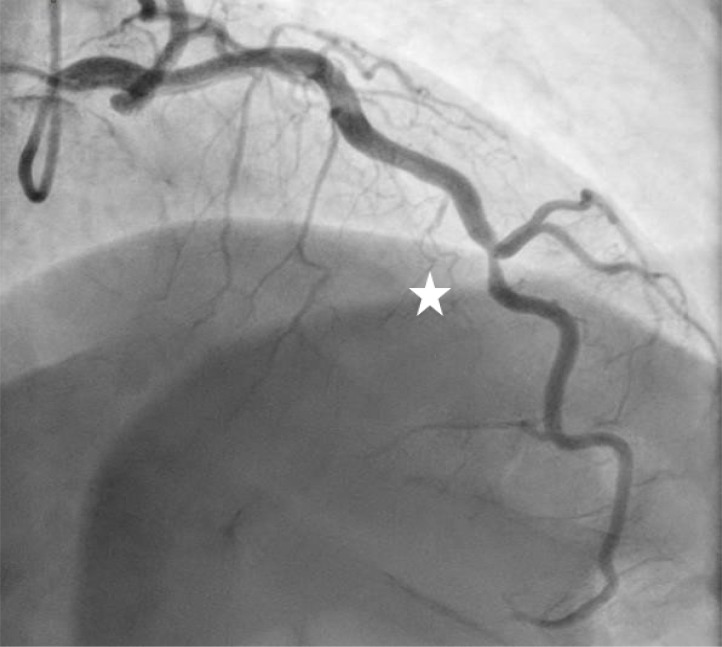
**Type 3 anterior descending SCAD (white star): the patient developed acute coronary syndrome (ACS) after repeated coughing during an asthma exacerbation with syncope**. SCAD, spontaneous coronary artery dissection.

**Fig. 5.**
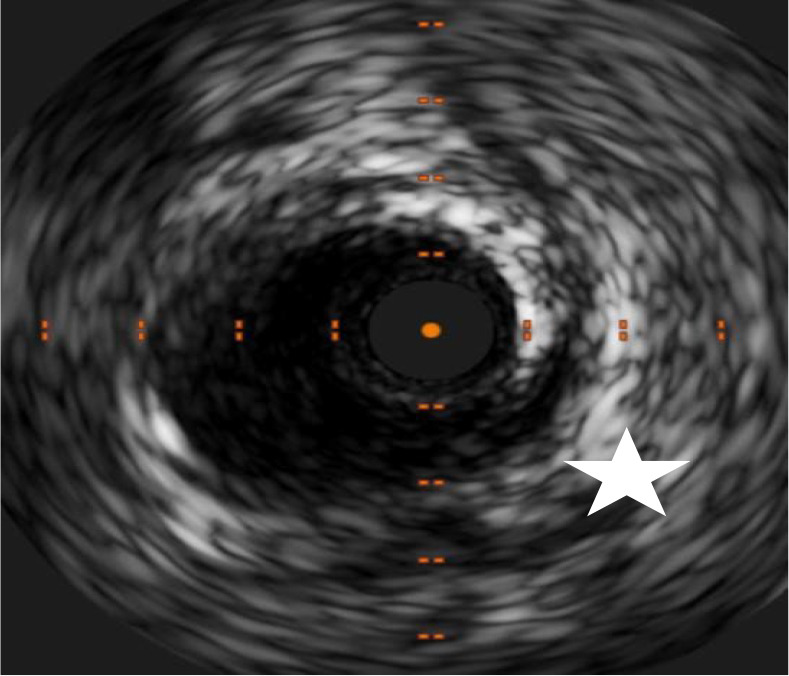
**Intimal tear (white star) and hematoma in the same patient from Fig. 4**.

**Fig. 6.**
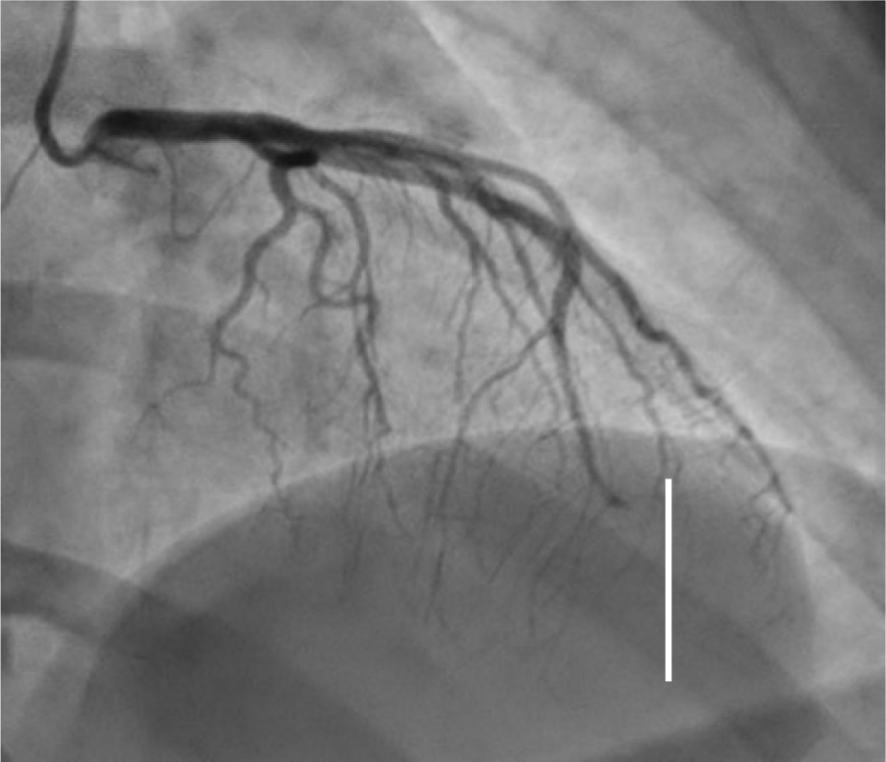
**Type 4 SCAD distal left anterior descending occlusion (white line)**. SCAD, spontaneous coronary artery dissection.

Regarding the execution technique of coronary angiography, there are no specific indications, except for more careful manipulation of the catheters and low flow contrast injection (to avoid exacerbating dissection) and use of intracoronary nitroglycerine to minimize the risk of spasm (since some SCAD presentations could be mistakenly interpreted as spasms) [15, 62]. The most frequently affected coronary artery is the left anterior descending (LAD), while the mid-distal segments are the most represented; meanwhile, multivessel involvement is present in 10% of cases [22, 63].

### 6.3 Intravascular Imaging (IVI)

IVUS and OCT are intravascular imaging techniques. They characterize the structure of the coronary and its layers, identifying elements such as intimal tears, hematomas of the media layer, fenestrations, and false lumens [31, 64]. Intravascular imaging is useful in doubtful cases, but it can also better define the mechanism of dissection, its starting and end points, and the involvement of bifurcation branches [64]. Being intravascular and invasive, IVUS and OCT risk enlarging and spreading the existing dissection. OCT, which requires an intracoronary contrast injection, must be used carefully, balancing risks and benefits [56, 64]. IVUS and OCT may guide the revascularization, confirming the correct guidewire position and defining the stenting extension [65]. Furthermore, imaging accurately defines the caliber (diameter and length), wall apposition, and positioning of the stent in the case of revascularization.

Since sizing a stent in a segment affected by SCAD is difficult, intravascular imaging is essential, although it risks malapposition (even late, when the hematoma reabsorbs).

**Table:** 

**ICA in SCAD golden rules**
- Extremely delicate catheter manipulation
- Use nitroderivate to discriminate between spasm and real lesion
- Minimize the injections (avoid dissection propagation)
- OCT and IVUS may clarify the diagnosis

## 7. ACS–SCAD-related Management

International guidelines on managing ACS advocate an early invasive strategy with revascularization of culprit lesions [53]. This applies to ACS in atherosclerotic disease: SCAD is different, and few data are available for treatment choice. The main differences with “normal” ACS are:

(1) In SCAD, medial dissection is the *primum movens* [66].

(2) PCI for SCAD is challenging and is associated with worse short- and long-term outcomes [67, 68, 69].

(3) Spontaneous healing is a frequent event [68].

(4) SCAD may be a recurrent event [67].

**Table:** 

**SCAD high-risk features**
- Recurrent chest pain
- Recurrent ST elevation
- Hemodynamic/electrical instability
- Left main/proximal lesions TIMI 0/1 flow

### 7.1 Treatment Rationale

The goals are myocardial damage reduction and recurrence prevention. The treatment approach depends on high-risk elements:

- Recurrent chest pain.

- Recurrent ST elevation.

- Hemodynamic instability, shock, or clinically significant ventricular arrhythmias.

- Involvement of multivessel severe proximal dissections or of the left main.

When high-risk features are present, consideration of immediate revascularization is warranted [42].

### 7.2 Conservative Management

Observational data have indicated angiographic “healing” of SCAD lesions in most patients (70%–97%) over a few months [69, 70, 71]. A minority of patients had persistent dissection on angiography [72]. Early complications may develop in 5% to 10% due to the extension of dissection within the first days after an acute episode. No clinical predictors of acute worsening have been identified. As for ACS, some days of hospital monitoring are needed [73, 74]. Nonetheless, ACS due to SCAD has lower mortality than ACS due to atherosclerosis, with a 3-year mortality incidence of 0.8% and a major adverse cardiovascular event (MACE) rate of 14.0%, driven largely by recurrent MI and unplanned revascularization [75]. Several systematic reviews and meta-analyses have examined long-term outcomes, comparing medical therapy vs. revascularization for SCAD, without differences between the two groups; however, no data are available after stratification according to high-risk features presence or in a randomized manner [75]. Interestingly, no difference between the groups was found in acute myocardial infarction (AMI) or SCAD recurrence (high risk vs. low risk).

**Table:** 

**Medical therapy in SCAD golden rules**
- AMI in SCAD remains AMI: reduce oxygen consumption (beta blockers, angiotensin-converting enzime (ACE)-inhibitor)
- vessel hematoma is predominant in SCAD: avoid dual antiplatelet therapy (DAPT)
- Atherosclerosis is not an issue in SCAD: statins only if indicated for other reasons

## 8. Revascularization in SCAD

Conservative management is the first choice in clinically stable patients [76]. In the presence of clinical high-risk features (recurrent chest pain/ST alteration, hemodynamic instability) or anatomic high-risk elements (left main involvement, multivessel SCAD, TIMI 0 flow), revascularization is the choice to consider [76]. If feasible, the preferable option is PCI over CABG [2, 76].

### 8.1 Percutaneous Coronary Intervention in SCAD

In SCAD, coronaries may present a weaker and altered structure, especially in the presence of systemic arteriopathies. PCI in SCAD may be challenging, considering the different pathophysiological substrates, with dissection extension, hematoma expansion risk, and late stent malapposition [67, 74, 77, 78]. Moreover, SCAD may affect distal segments with small caliber and tortuous courses, determining a low probability of intervention success [72, 73]. These technical complexities have been associated with adverse clinical outcomes in several series. In a Mayo Clinic series, PCI failed in 53% of the patients initially managed with PCI [5]. Similarly, in a European study of 134 patients, 27% of PCIs resulted in technical failure, and 9% of patients required emergency CABG [74]. Considering these elements, an optimized PCI approach is mandatory.

#### Optimizing PCI Approaches

**Fig. 7.**
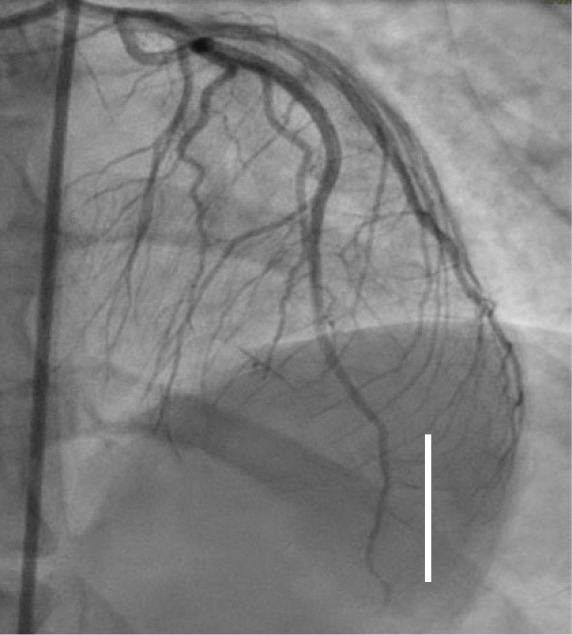
**Final result after wiring with SUOH 03 and percutaneous coronary intervention (PCI) (white line)**.

**Fig. 8.**
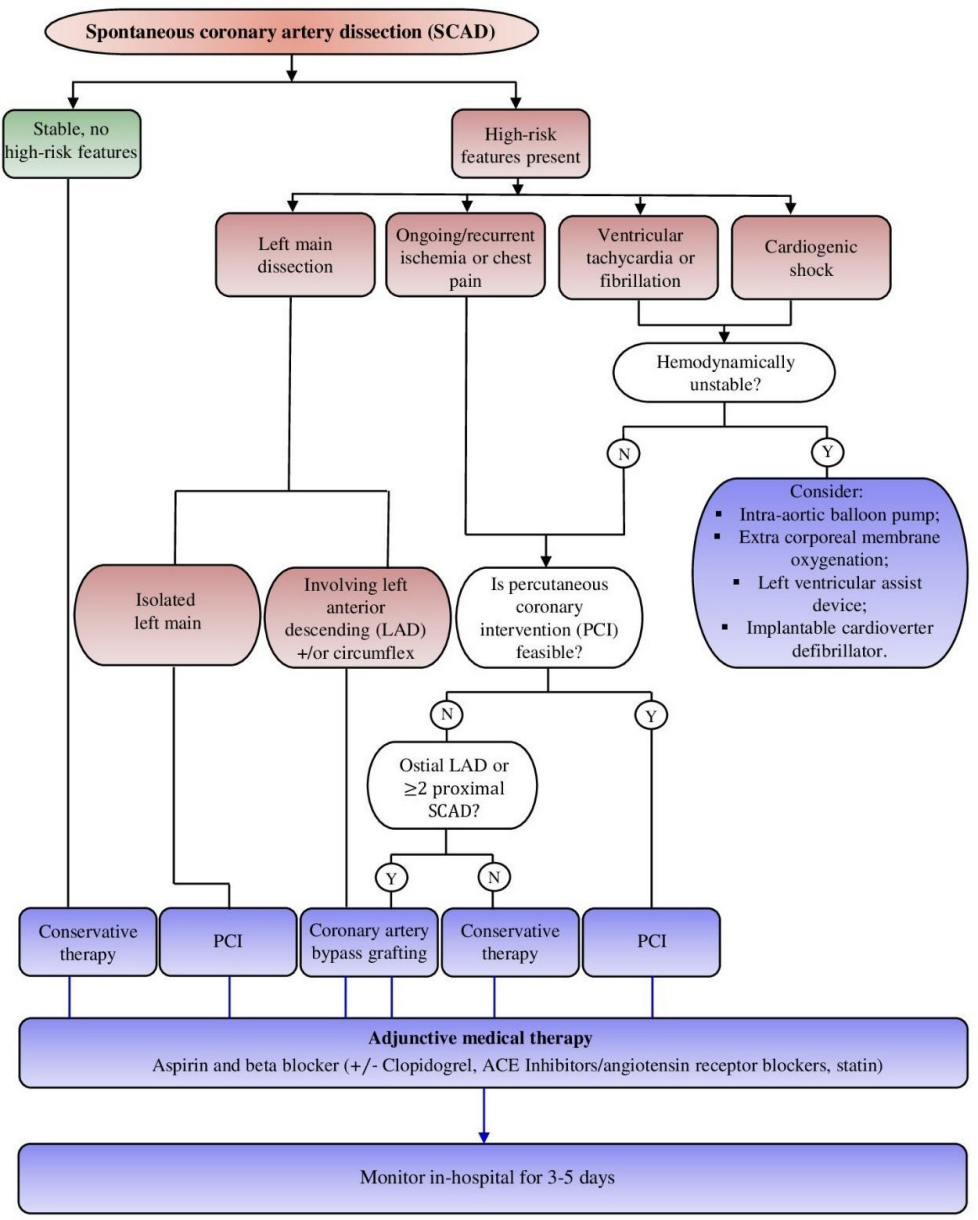
**SCAD management flow chart**. Y, yes; N, no, +/- Clopidogrel, clopdiogrel by clinician judgement; SCAD, spontaneous coronary artery dissection; ACE, angiotensin-converting enzime.

Multiple interventional strategies have been described when PCI is pursued for SCAD lesions. The main risk is the dissection propagation during the PCI attempt. To reduce this risk, first, avoid deep catheter engagement, noncoaxial positioning of the catheter tip, and contrast injections (to minimize mechanical or hydraulic propagation of dissection). With regard to lesion preparation and stent implantation techniques, several elements need to be considered. Starting from wiring, low tip load SUOH 0.3 “rope coil” wire (Asahi Intecc, Japan) is required to minimize the risk of flap enlargement and dissection propagation [79, 80]. In general, using polymer guidewires should be avoided due to the ease of engagement with the false lumen and consequent extension of the dissection. For stent implantation, selecting a stent length exceeding 5 to 10 mm on both proximal and distal edges of the dissection is advisable to accommodate propagation of the hematoma when compressed by the stent [24]. It is also preferable to avoid pre-dilation due to the risk of extension of the hematoma. Some advanced approaches are described in specific situations. For a reduction in occlusive hematoma, aspiration through microcatheter after sealing the entry site of dissection by stenting may reduce false lumen compression. This technique is called the “aspiration and sealing” technique [81]. In case of difficulty in regaining the true lumen, a double wire and double guiding catheter technique with realtime IVUS guidance to wire the true lumen and IVUS-assisted PCI has been described in iatrogenic ostial catheter-induced dissections but could be of some usefulness in PCI of SCAD. This technique requires extensive experience in complex/chronic total occlusion treatment [82]. In case of failure of other techniques, such as bailout, cutting balloon fenestration of the hematoma to decompress blood in the false lumen [83, 84]. Data on cutting-balloon angioplasty in SCAD remains limited [85, 86, 87]. Intravascular imaging (IVUS or OCT) is encouraged to help with stent sizing and position, reducing the risk of late malapposition; finally, after successful PCI, dual-antiplatelet therapy should be administered according to the stents implanted, as usual (Figs. 7,8).

**Table:** 

**The golden rules of PCI in SCAD**
- PCI is reserved for cases with high-risk characteristics
- Avoid polymer guidewires, preferring low-tip weight (SUOH 03)
- Minimize contrast injections (hydraulic dissection)
- Use imaging to confirm position and define stent size and apposition

### 8.2 Coronary Artery Bypass Grafting

CABG has been described as a treatment strategy for SCAD in patients with left main stem or proximal dissections after failed or not feasible PCI [71]. Further, follow-up data are not so encouraging during long-term follow-up, with a high rate of conduit failure, probably due to the healing of native SCAD vessels and competitive flow promoting graft occlusion. Given frequent late graft failure, the use of vein grafts may be considered, preserving arterial conduits for the future, but no data are available.

## 9. Thrombolysis

Systemic thrombolysis in SCAD is present only in case reports. Considering the risk of hematoma propagation and even coronary perforation, thrombolysis in SCAD is not indicated [6].

### SCAD and Cardiogenic Shock (CS)

Cardiogenic shock (CS) and ventricular arrhythmias may complicate SCAD, and the incidence of CS in SCAD is between 1.2% and 15.9% [11, 88, 89]. Patients with CS are usually younger, and they are more likely to have connective tissue disorder and grand multiparity and to be peripartum [90]. CS treatment in SCAD follows the well-known rules from the CS ESC guidelines, with escalation up to mechanical support and heart transplant [52].

## 10. Long-Term Medical Management

The goals of short- and long-term medical therapy for SCAD are to alleviate symptoms, improve short- and long-term outcomes, and prevent recurrent SCAD.

### 10.1 Anticoagulation and Antiplatelet Therapy

Owing to the pathophysiology, mechanisms of ischemia, and PCI outcomes for SCAD being distinct from those associated with atherosclerotic ACS, it is unclear if standard antiplatelet/anticoagulation therapy is beneficial. For instance, early heparin use may benefit by reducing the thrombus burden; however, theoretical concerns about its use in acute SCAD presentation are related to accentuating the risk of bleeding into the IMH or extension of dissection. Similarly, no data are available to guide the use of glycoprotein IIb/IIIa inhibitors in the emergency management of SCAD [23]. The benefits of early dual antiplatelet therapy (DAPT) in SCAD include protection from additional thrombosis in the prothrombotic environment caused by intimal dissection, but there is no clear data on this. Some observational data comes from a retrospective analysis of 1-year outcomes of SCAD patients, with the incidence of MACEs significantly higher in those treated with DAPT than SAPT [50, 91]. Considering the increased bleeding risks with antiplatelet agents, especially menorrhagia in premenopausal women, and uncertain benefits and risks, individual selection of suitability for aspirin therapy in conservatively managed survivors of SCAD is indicated [50, 91, 92].

### 10.2 β-Adrenergic Blockers

β-blockers should be considered in patients with SCAD who have ventricular dysfunction or arrhythmias and for the management of hypertension. However, in a recently reported 327-patient series from Vancouver with a median duration of follow-up of 3.1 years, the use of β-blockers was associated with a lower risk of recurrent SCAD in a multivariable analysis, a finding that strengthens the practice of β-blocker administration after SCAD [63].

### 10.3 Statins

Statin therapy is not recommended routinely after SCAD but is reserved for patients meeting guideline-based indications for primary prevention of atherosclerosis and for the management of patients with established concomitant atherosclerotic disease or diabetes mellitus.

### 10.4 Antianginal Therapy

Chest pain after SCAD is common and is a frequent cause of hospital readmissions. It accounts for 20% of readmissions within 30 days after acute myocardial infarction due to SCAD [9]. Symptoms may continue for several months after acute myocardial infarction even if evaluations of ischemia are normal or repeat coronary imaging shows vessel healing [93]. Chest pain in patients with abnormal ischemia testing should be treated with medical therapy and investigated with further cardiac testing. However, coronary vasospasm, endothelial dysfunction, microvascular disease, catamenial chest pain, and noncardiac chest pain should be considered in patients who continue to have atypical chest pain that is not associated with abnormal ischemia testing. Nitrates, calcium-channel blockers, and ranolazine are potential therapies to consider [56].

## 11. Follow-up

After SCAD, a clinical follow-up is indicated, such as for ACS. Additionally, at least 30 days after, non-invasive angiographic control (CCTA) may be recommended [6]. Total body CT (if not performed at the index event) is indicated to detect extracardiac vascular anomalies [6, 71].

### 11.1 Prevention of SCAD Recurrence

The risk of mortality in the follow-up of SCAD is low, around 2% at one year and 1% between 2 and 3 years [19]. Meanwhile, the risk of recurrent heart attacks is significant (up to 18% at 4 years) [23, 71]. In particular, the recurrence of events is often due to a recurrence of SCAD, but in a different location of the same coronary artery or even in a different coronary artery than the one affected by the initial event (Hayes *et al*. [2]). Some factors seem to correlate with recurrent SCAD: Hypertension, fibromuscular dysplasia, migraines, and coronary tortuosity [19]. Initial data support the use of beta-blockers in this context [94]. Although intense physical exertion can trigger SCAD, there are no clear indications regarding limitations on physical activity, although dedicated rehabilitation programs are hypothesized [93]. Generally, isometric efforts and activities requiring the Valsalva maneuver are discouraged [2]. There is greater uncertainty about SCAD recurrence during pregnancy. In the case of planned pregnancy in a patient with previous SCAD, it is necessary to inform the patient more adequately about the risks [2].

### 11.2 Chest Pain in SCAD Survivors

An increased incidence of chest pain is described in the follow-up of patients surviving SCAD. Considering the risk of recurrences, it is important to evaluate patients suspected of SCAD through clinical assessment, laboratory tests, ECG, and echocardiography. A new coronary angiography is indicated only in the presence of high clinical suspicion (such as positive troponin), given the risk of possible iatrogenic dissections. In this context, CCTA is the preferred examination [6].

### 11.3 Pregnancy in SCAD Survivors

Data on pregnancies post-SCAD are extremely limited. Therefore, recommendations are based on classic attention elements (such as left ventricular dysfunction symptoms). However, close follow-up during pregnancy is advisable [6, 15].

### 11.4 Psychological Behaviour

Psychological elements such as anxiety and depression are often already present in patients with SCAD, often further exacerbated after the acute event, leading to clear post-traumatic disorder and a decreased quality of life (Johnson *et al*. [95], 2020). For this reason, psychological assessment is recommended, thus reducing risk factors for further SCAD [95].

### 11.5 Post-SCAD Support and Education

To accelerate the return to normalcy for survivors of SCAD, as well as being a source of information and education for both families and the general population, the SCAD Alliance was created. Information on the reference website SCADalliance.org is available regarding both the nature of the SCAD and its clinical management.

## 12. Conclusions

SCAD is a relatively rare condition but with significant clinical consequences. From an epidemiological standpoint, it presents an asymmetrical distribution between genders and age groups, accounting for up to 20% of ACS in women under 50 years of age, according to some estimates. In its etiopathogenesis, the central moment is the separation of intima from media within the arterial wall due to intramural hematoma formation, with true lumen compression and blood flow hindrance. Although clinical presentation may include all classical signs and symptoms typical of ACS, SCAD diagnosis is not easy due to the low/atypical risk profile for ACS in patients affected. In these patients, the diagnostic hypothesis of SCAD should be considered, especially in the presence of predisposing or risk factors. An early diagnosis and appropriate treatment positively impact the prognosis and quality of life after SCAD. Regarding the therapeutic approach, some uncertainties concern the optimal antiplatelet therapy for acute management and recurrence prevention. However, there is more certainty on the indication for revascularization (almost always percutaneous): only in the presence of high-risk clinical and anatomical elements is PCI indicated (with complex procedures skills), while in other conditions, conservative treatment is the best choice, allowing for spontaneous healing of the dissection.

In conclusion, evidence regarding SCAD diagnosis and treatment is growing, but the primary recommendations come from retrospective registries and consensus documents. Soon, we hope that data of greater validity will emerge to confirm current clinical practices.
